# Obesity surgery makes patients healthier and more functional: real world results from the United Kingdom National Bariatric Surgery Registry^☆^

**DOI:** 10.1016/j.soard.2018.02.012

**Published:** 2018-07

**Authors:** Alexander Dimitri Miras, Anna Kamocka, Darshan Patel, Simon Dexter, Ian Finlay, James C. Hopkins, Omar Khan, Marcus Reddy, Peter Sedman, Peter Small, Shaw Somers, Suzie Cro, Peter Walton, Carel W. le Roux, Richard Welbourn

**Affiliations:** a Imperial College London, Division of Diabetes, Endocrinology and Metabolism, Hammersmith Hospital Campus, London, United Kingdom; b Leeds Teaching Hospitals, West Yorkshire, United Kingdom; c Royal Cornwall Hospital, Truro, United Kingdom; d Southmead Hospital, Westbury-on-Trym, Bristol, United Kingdom; e St. George’s University Hospital, London, United Kingdom; f Hull and East Yorkshire Hospital, Hull, United Kingdom; g Sunderland Hospital, Sunderland, United Kingdom; h Queen Alexandra Hospital, Portsmouth, United Kingdom; i Imperial College London, Imperial Clinical Trials Unit, London, United Kingdom; j Dendrite Clinical Systems Ltd, The Hub, Henley-on-Thames, United Kingdom; k Diabetes Complications Research Centre, Conway Institute, University College Dublin, Belfield, Dublin, Ireland; l Department of Upper GI and Bariatric Surgery, Musgrove Park Hospital, Taunton, United Kingdom

**Keywords:** Obesity, Bariatric surgery, Metabolic surgery, Diabetes, Functional status, Surgical registry

## Abstract

**Background:**

The National Bariatric Surgery Registry (NBSR) is the largest bespoke database in the field in the United Kingdom.

**Objectives:**

Our aim was to analyze the NBSR to determine whether the effects of obesity surgery on associated co-morbidities observed in small randomized controlled clinical trials could be replicated in a "real life" setting within U.K. healthcare.

**Setting:**

United Kingdom.

**Methods:**

All NBSR entries for operations between 2000 and 2015 with associated demographic and co-morbidity data were analyzed retrospectively.

**Results:**

A total of 50,782 entries were analyzed. The patients were predominantly female (78%) and white European with a mean age of 45 ± 11 years and a mean body mass index of 48 ± 8 kg/m^2^. Over 5 years of follow-up, statistically significant reductions in the prevalence of type 2 diabetes, hypertension, dyslipidemia, sleep apnea, asthma, functional impairment, arthritis, and gastroesophageal reflux disease were observed. The "remission" of these co-morbidities was evident 1 year postoperatively and reached a plateau 2 to 5 years after surgery. Obesity surgery was particularly effective on functional impairment and diabetes, almost doubling the proportion of patients able to climb 3 flights of stairs and halving the proportion of patients with diabetes related hyperglycemia compared with preoperatively. Surgery was safe with a morbidity of 3.1% and in-hospital mortality of .07% and a reduced median inpatient stay of 2 days, despite an increasingly sick patient population.

**Conclusions:**

Obesity surgery in the U.K. results not only in weight loss, but also in substantial improvements in obesity-related co-morbidities. Appropriate support and funding will help improve the quality of the NBSR data set even further, thus enabling its use to inform healthcare policy.

The increasing prevalence of obesity in the United Kingdom (U.K.) is well known and remains depressing. It is less well recognized that body mass index (BMI) on its own is a poor predictor of disease burden at the individual level and of mortality at the population level [1]. In epidemiologic studies the medical, functional, and psychological complications of obesity are the best predictors of mortality after 16 years of observation [1].

Large case-control studies and randomized controlled trials (RCTs) have shown that bariatric surgery reduces obesity-related morbidity and/or mortality from conditions such as cancer [2], obstructive sleep apnea [3], cardiovascular disease [4], type 2 diabetes (T2D) microvascular complications [5], functional impairment [6], infertility [7], and nonalcoholic fatty liver disease [8]. Several registries, such as the International Federation for the Surgery of Obesity and Metabolic Disorders (IFSO) Global Registry [9] and the Scandinavian Obesity Registry [10], have been also consistent in showing safety and efficacy of obesity surgery on these co-morbidities. Thus, bariatric surgery has evolved from an intervention purely for weight, to one for diabetes control and ultimately for obesity as a “systems disease.”

The uptake of obesity surgery in the U.K. has been slower and more cautious compared with other parts of the world. In an attempt to collect data on disease burden of the operated population and to increase the openness and transparency of surgery outcomes, the British Obesity and Metabolic Surgery Society in collaboration with the Association of Laparoscopic Surgeons of Great Britain and Ireland and the Association of Upper Gastrointestinal Surgeons and in partnership with Dendrite Clinical Systems Limited launched the National Bariatric Surgery Registry (NBSR) in January 2009. It became mandatory to submit National Health Service (NHS) England data to the NBSR and publish surgeon-level outcomes in 2013. Unlike the NBSR, independently collected Hospital Episode Statistics or community databases do not collect systematic data on patient demographic characteristics or disease burden. Our aim was to determine whether the effects of obesity surgery on associated co-morbidities from RCTs could be replicated in a “real life” setting within the U.K. health service. We extracted data entries from the NBSR to examine the disease burden of 50,782 patients and the effects of obesity surgery on related co-morbidities up to 5 years postoperatively.

## Methods

The first 3756 entries in the NBSR (7.4%) from year 2000 to 2008 were entered retrospectively, and all entries from February 2, 2008 to January 12, 2015 were entered prospectively and analyzed retrospectively. The NBSR software is a bespoke web registry application built by Dendrite Clinical Systems using their Intellect Web proprietary software, hosted on a secure server within the NHSNet *N3* network. Surgeons performed most data entry, with some by specialist nurses/dieticians and obesity physicians. Two hundred six surgeons from 159 hospitals contributed to the current data set. Patient consent was not obtained because these were routinely generated clinical data. All entries are anonymized to comply with the data governance regulations, and data collection for this analysis was performed in compliance with the Declaration of Helsinki.

The variables used in the analysis include baseline demographic data (patient’s age, sex, ethnic background), weight, height, BMI, Edmonton Obesity Staging System (EOSS), and obesity-related co-morbidities (T2D and its duration preoperatively, hypertension, dyslipidemia, sleep apnea, asthma, functional impairment, arthritis, gastroesophageal reflux disease, liver disease, polycystic ovary syndrome [PCOS], cardiovascular disease, depression, venous thromboembolism status) and changes in their prevalence over the 5-year follow-up (Table 1, Table 2). Surgical and perioperative data comprised the type of procedure, its mode (primary versus revision), approach (laparoscopic versus open versus endoscopic), American Society of Anesthesiologists grade, Obesity Surgery Mortality Risk Score, morbidity, and mortality (Table 3). The EOSS has been used to assess and stage obesity-related co-morbidity better. In brief, it assesses mechanical, metabolic, and psychological aspects of obesity and separates patients into 5 risk groups: no clear risks (stage 0), preclinical (stage 1), established risks or disease (stage 2), end-organ damage (stage 3), and end-stage disease (stage 4). We used the co-morbidity data recorded in the NBSR data set to assign an approximate EOSS score (Supplemental Table 1). It was beyond the scope of this analysis to compare the effectiveness of the different obesity surgery procedures on these variables.

**Table 1:** Baseline characteristics

Variable	Mean ± SD [range] or (percentage)
Age	45 ± 11 [12–84]
Sex	
Male	22.2%
Female	77.8%
Ethnicity	42,806
Caucasian	90.4%
Afro-Caribbean	2.3%
African	1.4%
Asian	3.2%
Other	2.7%
Weight, kg	121.7 ± 43.5 [46.0–213.8]
Height, m	1.66 ± .09 [1.35–1.96]
BMI, kg/m^2^	47.8 ± 8.0 [25.0–73.7]

SD = standard deviation; BMI = body mass index.

**Table 2:** Co-morbidities recorded at baseline

Variable	
Edmonton Obesity Staging System	
0	5.7
I	11.6
II	55.1
III	11.5
IV	16.1
T2D status	
No indication of T2D	71.3
Impaired glycaemia or impaired glucose tolerance	4.4
Oral hypoglycemics	17.3
Insulin treatment	7
T2D duration	
≤5 yr	59.2
6–10 yr	22.4
>10 yr	18.4
Hypertension status	
No indication of hypertension; or on no treatment	63.2
Hypertension on treatment	36.8
Dyslipidemia status	
No indication of dyslipidemia	78
Dyslipidemia	22
Cardiovascular disease status	
No indication of atherosclerosis	94.8
Diagnosed atherosclerosis	5.2
Sleep apnea status	
No diagnosis or indication of sleep apnea	79.8
Diagnosis of sleep apnea; on CPAP/BiPAP	19.7
Sleep apnea with complications	0.5
Asthma status	
No diagnosis or indication	81
Treated with inhalers	17.5
Treatment with nebulizers or oral steroids; or requiring hospital admission in last year	1.5
Impaired functional status	
Can climb 3 flights of stairs without resting	30.3
Can climb 1 flight of stairs without resting	48.2
Can climb half a flight of stairs without resting	18.6
Requires wheelchair/housebound	2.9
Venous thromboembolism status	
No known risk factors	80
History or risk factor for DVT/PE	6
Obesity/hypoventilation syndrome	13.5
Venous edema with ulceration	0.4
Vena cava filter	0.1
Arthritis status	
No symptoms	46.5
Intermittent symptoms; no medication	22.4
Regular medication with nonopiates	19.4
Known arthritis/requiring opiates	8.5
Back/joint operation done/recommended pending weight loss	2.9
Failed previous back operation/joint replacement	0.3
Liver disease	
No indication of liver disease	94.5
Suspected NAFLD	3.6
Known NAFLD	1
NASH	0.6
Cirrhosis	0.3
GERD	
No symptoms	64.7
Intermittent symptoms; no medication	13.4
Intermittent medication	6.1
Daily medication: H2 RA/PPI	15.7
Previous antireflux operation	0.1
PCOS	
No indication/diagnosis; no medication	90.5
Diagnosis of PCOS; no medication	5.1
PCOS on medication	3.8
Infertility	0.6
Depression	
No indication of depression	73.8
Depression on medication	26.2

T2D = type 2 diabetes; CPAP = continuous positive airway pressure; BiPAP = bilevel positive airway pressure; DVT = deep vein thrombosis; PE = pulmonary embolism; NAFLD = nonalcoholic fatty liver disease; NASH = nonalcoholic steatohepatitis; GERD = gastroesophageal reflux disease; H2 RA = H2 receptor antagonist; PPI = proton pump inhibitor; PCOS = polycystic ovary syndrome.

**Table 3:** Operative information

	
Type of operation	
Roux-en-Y gastric bypass	51.4
Vertical sleeve gastrectomy	20.2
Gastric banding	19.7
Revisional gastric banding surgery	2.5
Gastric balloon placement/removal	2.8
Duodenal switch	0.2
Duodenal switch with sleeve	0.1
Bilio-pancreatic diversion	0.1
Other	3.0
Mode of surgery	
Primary	90.4
Revision	2.2
Revision as a primary procedure (in your hands)	5.1
Planned second stage	2.3
Procedure approach	
Laparoscopic	92.7
Open	4.5
Endoscopic	2.4
Laparoscopic converted to open	0.4
ASA	
I	13.6
II	61.3
III	24.6
IV	0.5
OSMRS	
A (lowest risk, 0–1)	50.9
B (intermediate risk, 2–3)	42.5
C (high risk, 4–5)	6.6
Perioperative Morbidity	3.1
Perioperative Mortality	0.07

ASA = American Society of Anesthesiologists; OSMRS = obesity surgery mortality risk score.

The vast majority of variables were recorded in a categorical manner. No standardized clinical definitions or laboratory ranges were used to define obesity-related co-morbidities; therefore, all entries were dependent on the judgment of the clinician submitting the data. Follow-up appointments were done at variable points, and therefore to standardize time-interval reporting only the closest data entry to the specific time points was included and all additional entries were excluded from the analysis. Complications, including death and reoperation, were recorded during the inpatient stay, within 30 days or at any time after surgery.

To systematically identify erroneous entries (i.e., height or weight), the robust regression and outlier removal method was applied. The robust nonlinear regression method was used to fit a curve not influenced by outliers, and the residuals of the robust fit then were analyzed to identify outliers. These were subsequently removed, and an ordinary least-squares regression on the remaining data was performed. The Q value, which determines how sensitively the potential erroneous entries are identified, was set to 10%.

GraphPad Prism version 7 (GraphPad Software, La Jolla, CA, USA), was used for statistical analysis. Data are presented as absolute values, percentages, mean ± standard deviation, or median (25th–75th percentile). D’Agostino-Pearson Omnibus normality test was used to assess normality distribution. Nonnormally distributed continuous variables were compared over time using the Kruskal-Wallis test. Postbaseline categorical variables were compared with baseline values using the χ^2^ test with Yates’ continuity correction. Longitudinal continuous data were analyzed with a repeated measures one-way analysis of the variance with Dunnett’s correction for multiple comparisons. Sensitivity analysis was conducted to assess the impact of missing data, using the primary analysis methods, but under alternative missing data assumptions. Statistical significance was defined as *P*<.05.

## Results

### Baseline characteristics

**Fig. 1:**
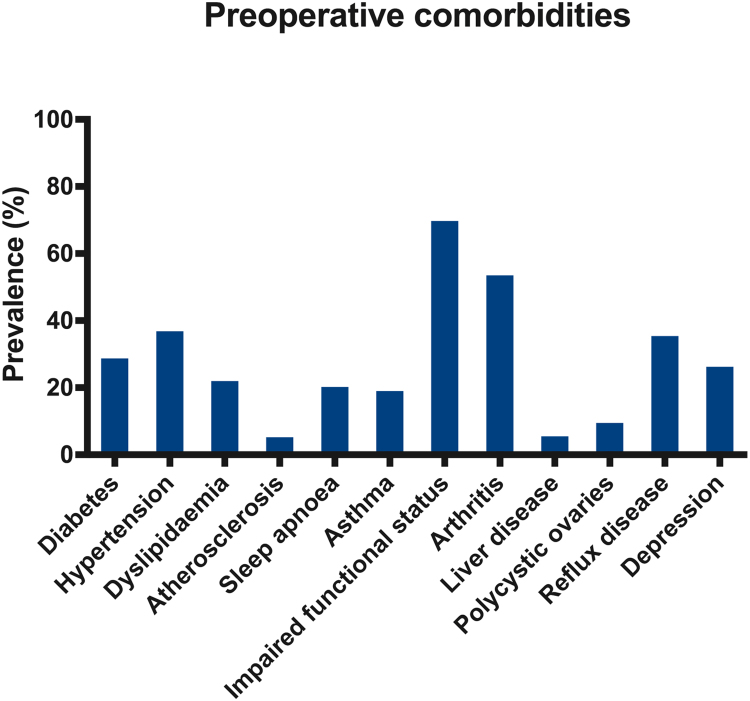
Obesity-related co-morbidities at baseline.

**Fig. 2:**
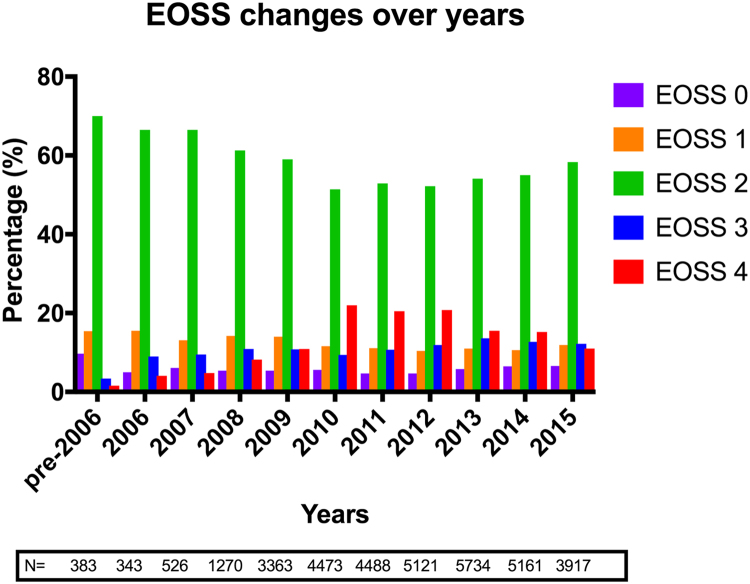
Edmonton Obesity Staging System score at baseline.

From February 2000 to December 2015, 50,782 procedures were recorded in the NBSR. The cohort consisted predominantly of middle-aged female patients of Caucasian ethnic background (Table 1). The commonest recorded co-morbidity was functional impairment (70%), followed by arthritis (54%) and hypertension (37%). Approximately 1 in 3 patients had T2D (Table 2; Fig. 1). A gradual increase in the median number of reported preoperative co-morbidities was observed over 15 years, with the median number of co-morbidities per patient rising from 1 before the year 2006, to 2 in 2006 to 2008, to 3 from 2009 onward (Kruskal-Wallis test, *P*<.001). The distribution of EOSS scores over 15 years is shown in Fig. 2.

### Health outcomes

**Fig. 3:**
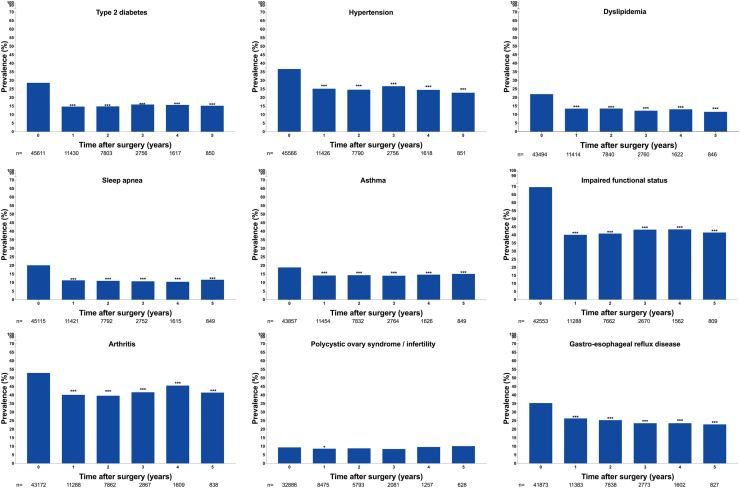
Obesity related co-morbidities at baseline and 5-year follow-up. *P* values as compared to the baseline: *P<.05, **P<.01, ***P<.001.

Over the 5-year follow-up, statistically significant reductions were observed in the prevalence of T2D (Fig. 3), hypertension, dyslipidemia, sleep apnea, asthma, functional impairment, arthritis, and gastroesophageal reflux disease. The “remission” of these co-morbidities was evident 1 year postoperatively and reached a plateau 2 to 5 years after surgery. Obesity surgery was particularly effective on functional impairment and T2D, with almost a doubling of the proportion of patients able to climb 3 flights of stairs compared with preoperatively and a halving of the proportion of patients with T2D-related hyperglycemia compared with preoperatively. The only recorded co-morbidity for which no significant change was recorded was PCOS and/or infertility (Fig. 3).

### Weight loss

**Fig. 4:**
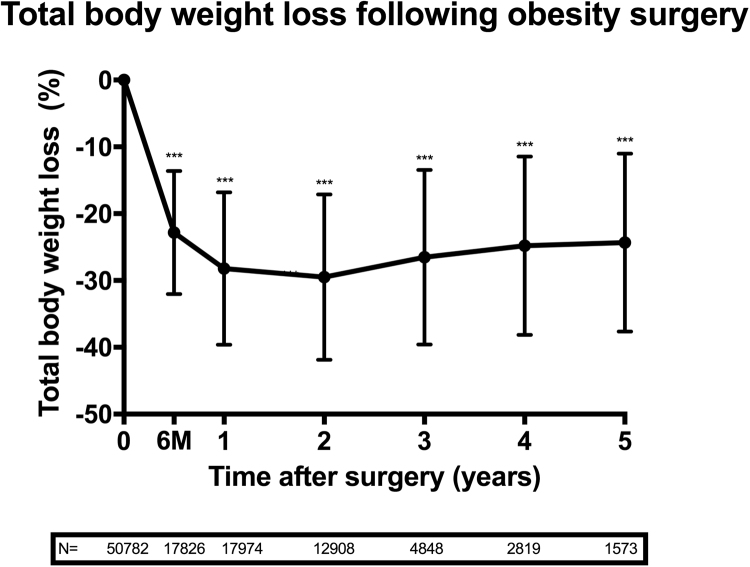
Weight loss after obesity surgery. Error bars represent standard deviation (SD). *P* values as compared to the baseline: *P<.05, **P<.01, ***P<.001.

The most rapid weight loss took place during the first year after surgery and reached a nadir of 30 ± 12% total weight loss at 2 years. This was followed by weight regain and 24 ± 13% total weight loss at 5 years (Fig. 4).

### Choice of surgery

**Fig. 5:**
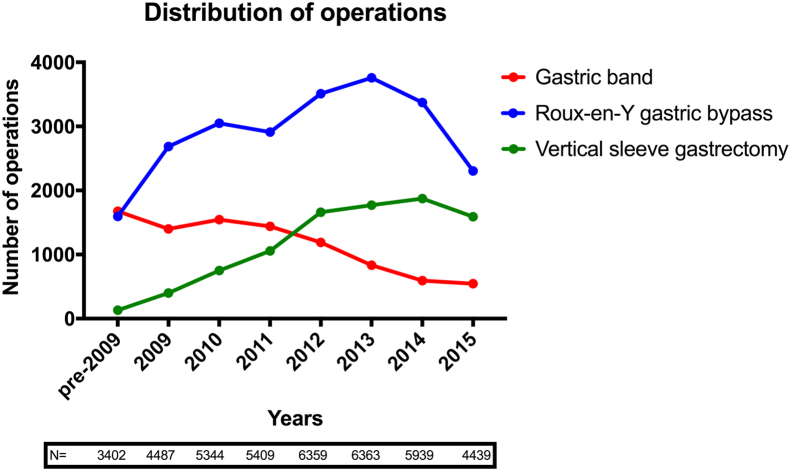
Trends in the type of obesity surgery procedures performed.

The most commonly performed operation was Roux-en-Y gastric bypass (RYGB; 51.4%), followed by vertical sleeve gastrectomy (VSG; 20.2%) and gastric banding (19.7%; Table 3). Over the course of the observation period, the percentage of RYGB procedures performed increased and reached a plateau. There was a decrease in the percentage of gastric banding and an increase in the percentage of VSG operations performed (Fig. 5). The remaining 8.7% of procedures included duodenal switch, biliopancreatic diversion, revisional gastric band, and intragastric balloon or were not specified. Most procedures were primary surgery (90%) and were performed laparoscopically (93%). Median postoperative length of stay was 3 days before 2006 and 2 days from 2007 onward.

### Postoperative complications and mortality

Recorded postoperative cardiovascular complications, such as dysrhythmia or myocardial infarction, were recorded in 0.3% patients. The total postoperative complications rate was reported as 3.1%, the most common being vomiting/poor oral intake (Supplemental Table 2). Recorded in-hospital mortality rate was 0.07%.

### Missing data

At baseline, the domain with the highest rates of incomplete entries (17.4%) was gastroesophageal reflux disease, followed by PCOS/infertility (16.8%). Data entry for all health outcomes decreased substantially after the first postoperative year (Fig. 3). The primary analysis was conducted under the assumption that the missing data were Missing Completely at Random (i.e., for each co-morbidity the prevalence in the unobserved patients was assumed to be the same as for those that were observed). Sensitivity analysis was conducted to examine the robustness of the purported significant results. For each co-morbidity, the number of patients required to overturn the significance of the observed result was established. Considerably higher proportions of co-morbidities were required among the unobserved than were seen in the observed to change the significance of the results. The results therefore support the primary conclusion of significant reductions in these co-morbidities postsurgery (Supplementary Table 3).

## Discussion

Using real-life data, we found that surgery reduces weight and improves health. Obesity-related physical and functional conditions were either ameliorated or even entered “remission” within 1 year after surgery, and this effect was durable at 5 years. These health benefits were achieved in the context of an increasingly “sick” patient population, with a reduction in the length of inpatient stay and with low rates of surgery-related morbidity and mortality.

The most striking improvements were observed in patients with impaired functional status or T2D at baseline with “resolution” in 58% and 52%, respectively, at 1 year postoperatively. The proportion of patients who could climb only half a flight of stairs at baseline reduced from 19% to 8% 1 year after surgery. Obese patients have higher rates of unemployment compared with normal weight individuals and functional impairment is one of the major contributors. Studies from the United Kingdom and other European countries demonstrated that 14 months after obesity surgery patients’ paid working hours increased by 57% and state benefit claims decreased by 75% [11], [12]. Indeed, the Office of Health Economics in the United Kingdom has predicted that if 25% of eligible patients for obesity surgery underwent surgery, the U.K. gross domestic product would increase by £1.3 billion due to the expected increases in paid employment, which would offset the costs of surgery within 1 year [13].

Patients with T2D experience rapid improvements in blood glucose, and 48% have no reported indication of diabetes within 1 year after obesity surgery. This has been shown to translate to less medication usage, fewer capillary glucose testing strips, fewer hospital encounters [14], [15], and—together with improvements in other cardiometabolic risk factors (e.g., hypertension and dyslipidemia)—into reduction in the incidence of T2D-related micro- and macrovascular complications [5], [16]. What is not reported in the NBSR are outcomes of T2D patients who continue on glucose-lowering medications postoperatively; many patients, despite not being able to stop all of these medications, still reduce their total number and manage to achieve optimal glycemic control postoperatively. This is still a very important but underreported outcome of obesity surgery. The IFSO Global Registry 2017 reports 63% patients coming off glucose-lowering medications 1 year after surgery. In the Scandinavian Obesity Registry this number reaches 66% after 1 year and 54% after 5 years [10]. Somewhat higher rates of reported T2D “remission” or stopping glucose-lowering medications in those registries might differ due to varying criteria between countries and bariatric centers when diagnosing T2D, withholding medications, or claiming remission.

The surprising lack of an effect on PCOS/infertility is contrary to the published literature [17]. It may be because a diagnosis of PCOS is not frequently reviewed, unlike T2D or hypertension, which are subject to constant reassessment. Furthermore, a significant proportion of women undergoing obesity surgery were postmenopausal; therefore; effects of surgery on improving fertility would not be monitored in that group.

Choice of surgery is somewhat different to that reported worldwide. In both the IFSO Global Registry 2017 and NBSR, RYGB is the most commonly performed procedure, constituting 46.3% of all operations as per IFSO records and 51.4% in the NBSR. However, reported numbers of VSG worldwide are almost as high as the RYGB (43.6%), while in the United Kingdom only 1 in 5 bariatric procedures was VSG. Gastric band numbers differ even further with only 6% by IFSO and 19.7% by the NBSR. Some variations in the proportion of each procedure can be explained by the fact that the latest IFSO Registry covers 2013 to 2017; the U.K. data presented here cover 15 years before 2015, and these are the years when VSG was gaining popularity and the gastric band procedure was declining. Furthermore, there are clear region-based trends regarding the procedure performed. European surgery preferences are similar to the United Kingdom, whereas Australasia, Central and South America, as well as the Middle East present VSG as their procedure of choice.

Performing obesity surgery in dedicated bariatric centers, along with use of laparoscopic techniques, enables low mortality and morbidity rates and short postoperative stays to be achieved. Even if there is underreporting of complications and postoperative deaths (e.g., due to loss of follow-up, when patients change healthcare providers), the 0.07% mortality and overall 2.6% complication rate rank obesity surgery as one of the safest major elective surgical procedures. The low in-hospital mortality reported in the NBSR is consistent with the Hospital Episode Statistics mortality data collected in the NHS in England [18].

The major benefit of the NBSR is that its real-life data complement the findings of RCTs of much smaller sample size. If combined appropriately with other large data sets, “big data” can provide invaluable information in terms of patient prioritization for surgery, personalization of treatment, and health economic analyses. An additional benefit is that the NBSR has already changed healthcare policy in the United Kingdom. In the context of limited available evidence from relevant RCTs on the benefits of surgery on patients with T2D duration of<2 years, the National Institute of Healthcare Excellence examined the NBSR data, which showed that being underinclusive would exclude approximately 75% of eligible patients receiving bariatric surgery. As a result, National Institute of Healthcare Excellence extended the definition of recent-onset T2D to include patients with disease duration of 10 years and a BMI of 30 to 35 kg/m^2^. More RCTs were thus able to be included in their analyses, leading to lowering of the BMI eligibility criteria for surgery to 30 kg/m^2^ [19].

The NBSR data set does not allow health economic analyses to be generated. However, the Health Technology Assessment program has already demonstrated that the incremental cost-effectiveness ratio per quality adjusted life year of obesity surgery was between £2000 and £4000 for a patient with a BMI ≥40 kg/m^2^ and therefore financially favorable [20]. Currently,<1% of patients eligible on BMI criteria undergo obesity surgery in the United Kingdom [21]. The level 2b evidence from the NBSR together with grade B recommendations from the Health Technology Assessment, and data from the Clinical Practice Research Datalink [10] in combination could encourage local healthcare commissioners to increase the funding available for obesity surgery.

The major limitation of the NBSR is sparse record of follow-up after 2 postoperative years. NHS England commissions surgical providers for 2 years of follow-up, with further follow-up in primary care after this. There is currently no mechanism to capture data from primary care in the registry [21]. This is in contrast with the well-developed infrastructure that supports data collection for cancer treatment and survival.

After a comparison of baseline characteristics between those unobserved and observed at 1 and 5 years the Missing Completely at Random assumption was considered most plausible for the missing data in the analysis. However, as operating surgeons enter most of the data, it cannot be ruled out that there may be an underreporting bias for operative complications (excluding death) and an overreporting bias for the positive outcomes of surgery. The small proportion of retrospective entries between 2000 and 2008 is vulnerable to this bias. Also, the early or late complications of patients attending a unit other than the one at which they had surgery would not be captured. In addition, the data set does not contain specific fields for postoperative gallstone disease or internal hernias.

The NBSR did not standardize definitions of specific diagnoses (e.g., T2D, hypertension) and did not include continuous data (e.g., glycated hemoglobin, blood pressure), imaging, and other clinical investigation results or detailed medication usage. Therefore, all entries were dependent on the judgment of the clinician submitting the data, not necessarily on robust disease diagnosis and remission criteria. The data set did not contain other clinically important variables (e.g., number of medications, nutrient deficiencies, fractures, substance abuse, and suicidal attempts after the operation). The data set was chosen as a balance between collecting too much data (risking poor engagement and incomplete records) and collecting too little data to generate meaningful analysis. These limitations could potentially be overcome in the future through consensus on clinical definitions, inclusion of patient identifiable information, and infrastructure to collect follow-up data from primary care.

## Conclusions

The NBSR data demonstrate on a large scale that obesity surgery leads to weight loss and substantial improvements in obesity-related co-morbidities. Patients become healthier and more functional. Surgery is safe despite the patients having more obesity-related disease over time. Appropriate support and funding will help improve the quality of the NBSR data set even further, thus potentially increasing its effect on healthcare policy.

## Disclosures

*The authors have no commercial associations that might be a conflict of interest in relation to this article.*

## Footnotes

Supplementary data Supplementary data associated with this article can be found in the online version at http://dx.doi.org/10.1016/j.soard.2018.02.012.
