# Supplementary material for: Obesity surgery makes patients healthier and more functional: real world results from the United Kingdom National Bariatric Surgery Registry
Source: Surg Obes Relat Dis. 2018 Jul;14(7):1033–40. doi: 10.1016/j.soard.2018.02.012 (PMC6097875; doi:10.1016/j.soard.2018.02.012)
Supplement: Supplementary file 1 — Supplementary material [file mmc1.docx]

**Supplemental table 1: Scoring of the Edmonton Obesity Staging System (EOSS)**

| **EOSS stage** | **Comorbidity** |
| --- | --- |
| **4**  *One or more of the following characteristics* | Requires a wheelchair or is housebound |
|  | Has venous oedema with ulceration |
|  | Has had a vena cava filter |
|  | Obesity/hypoventilation syndrome |
|  | Liver cirrhosis |
|  | |
| **3**  *If no criteria for EOSS stage 4 are met, one or more of the following qualifies as EOSS 3* | Diagnosed with atherosclerosis |
|  | Sleep apnoea with complications |
|  | Asthma requiring treatment with nebulisers or oral steroids or requiring hospital admission in the last year |
|  | Known arthritis / back or leg pain from arthritis requiring opiates |
|  | Non-alcoholic steatohepatosis proven on liver biopsy |
|  | Infertility (female patients only) |
|  | |
| **2**  *If no criteria for EOSS stages 3 and 4 are met, one or more of the following qualifies as EOSS 2* | Oral hypoglycemic or insulin therapy for type 2 diabetes |
|  | Hypertension on treatment |
|  | Dyslipidemia |
|  | Diagnosis of sleep apnoea or on CPAP/BiPAP |
|  | Asthma treated with regular inhalers |
|  | Can climb half a flight of stairs without resting |
|  | Back or leg pain from arthritis requiring regular medication with non-opiates |
|  | Daily medication with H2RA/PPI or previous anti-reflux disease |
|  | A prior operation for reflux disease |
|  | Known non-alcoholic fatty liver disease proven on biopsy or hepatology opinion |
|  | Depression on medication |
|  | On medication for polycystic ovary syndrome (female patients only) |
|  | |
| **1**  *If no criteria for EOSS stages 2, 3 and 4 are met, one or more of the following qualifies as EOSS 1* | Impaired glycaemia or impaired glucose tolerance |
|  | Intermittent symptoms of back or leg pain from arthritis not treated with medication |
|  | Can climb 1 flight of stairs without resting |
|  | Suspected non-alcoholic fatty liver disease (abnormal liver function tests or abnormal ultrasound scan) |
|  | Diagnosis of polycystic ovary syndrome that is not treated with medication (females only) |
|  | Intermittent medications or intermittent symptoms of reflux disease |
|  | |
| **0**  *No criteria for EOSS staged 1-4 are met* |  |

EOSS - Edmonton Obesity Staging System (EOSS)
CPAP - Continuous Positive Airway Pressure
BiPAP - Bilevel Positive Airway Pressure
H2RA - H2 Receptor Antagonist
PPI - Proton Pump Inhibitor
