# Supplementary material for: Obesity surgery makes patients healthier and more functional: real world results from the United Kingdom National Bariatric Surgery Registry
Source: Surg Obes Relat Dis. 2018 Jul;14(7):1033–40. doi: 10.1016/j.soard.2018.02.012 (PMC6097875; doi:10.1016/j.soard.2018.02.012)
Supplement: Supplementary file 2 — Supplementary material [file mmc2.docx]

**Supplemental table 2: Complications recorded post-operatively**

| **Complication** | **N** | **Percentage (%)** |
| --- | --- | --- |
| Fluid / electrolyte problems | 219 | 0.4 |
| Acute cholecystitis / biliary colic | 2 | 0.0 |
| CBD stones / cholangitis | 2 | 0.0 |
| Gastric distention | 26 | 0.1 |
| Other abscess / infection / fever | 223 | 0.4 |
| Acute renal failure | 46 | 0.1 |
| Pneumonia / atelectasis | 237 | 0.5 |
| Rhabdomyolysis | 5 | 0.0 |
| Urinary tract infection | 36 | 0.1 |
| Vomiting / poor intake | 296 | 0.6 |
| Wound infection / breakdown | 156 | 0.3 |
| Unanticipated transfer to ITU | 152 | 0.3 |
| Cardiovascular complications | 155 | 0.3 |
| **Total** | **1555** | **3.1** |

CBD – common bile duct
ITU – Intensive Care Unit
