# Supplementary material for: Obesity surgery makes patients healthier and more functional: real world results from the United Kingdom National Bariatric Surgery Registry
Source: Surg Obes Relat Dis. 2018 Jul;14(7):1033–40. doi: 10.1016/j.soard.2018.02.012 (PMC6097875; doi:10.1016/j.soard.2018.02.012)
Supplement: Supplementary file 3 — Supplementary material [file mmc3.docx]

Table 4. Sensitivity analysis of health outcomes

| Health outcome | Prevalence at baseline | Prevalence at 1 yr | |
| --- | --- | --- | --- |
|  |  | Known prevalence in observed cases | Prevalence required in unobserved cases to shift result to insignificance (*P* > .05) |
| T2D | 28.7% | 14.8% | 32.7% |
| Hypertension | 36.8% | 25.3% | 37.2% |
| Dyslipidemia | 22.0%% | 13.5% | 24.3% |
| Sleep apnea | 20.2% | 11.4% | 22.5% |
| Asthma | 19.0% | 14.3% | 20.0% |
| Impaired functional status | 69.7% | 40.2% | 79.5% |
| Arthritis | 53.5% | 40.2% | 57.5% |
| GERD | 35.4% | 26.4% | 37.9% |

T2D = type 2 diabetes; GERD = gastroesophageal reflux disease.
